# Phytotherapy in treatment of Parkinson’s disease: a review

**DOI:** 10.1080/13880209.2019.1618344

**Published:** 2019-05-29

**Authors:** Zahra Rabiei, Kamal Solati, Hossein Amini-Khoei

**Affiliations:** Medical Plants Research Center, Basic Health Sciences Institute, Shahrekord University of Medical Sciences, Shahrekord, Iran

**Keywords:** Dopaminergic receptors, L-DOPA, *Ginkgo biloba*, curcumin

## Abstract

**Context**: Parkinson’s disease (PD) is a neurodegenerative disorder due to gradual loss of dopaminergic nerves in the substantia nigra (SN) in the midbrain. PD leads to certain motor disorders including resting tremor, muscle stiffness and slow movement. Medicinal plants have shown positive pharmacological effects in treating different models of PD.

**Objective:** Tendency to use natural products, especially plants, for the treatment of PD has been growing. This article reviews the basic aspects of medicinal plants and their bioactive compounds that could be used to treat PD.

**Methods:** Reliable articles indexed in databases ISI, SID, PubMed, PubMed Central, Scopus and Web of Science were used. A total of 12 plant-derived active ingredients and 18 herbal extracts were included. Different compounds have so far been isolated from plants that affect PD especially by targeting pathways associated with the pathogenesis of the disease.

**Results:** Although some herbal extracts such as *Hibiscus asper* Hook. f. (Malvaceae), *Ginkgo biloba* L. (Ginkgoaceae)*, Carthamus tinctorius* L (Asteraceae) and certain active ingredients, such as berberine and curcumin, have shown positive effects in animal models of PD, potential active ingredients and mechanisms of action should be investigated in additional studies.

**Discussion and conclusions:** Despite the wide variety of plants in the world, a limited number of them have been studied for anti-Parkinsonian activity, and therefore, there are numerous perspectives in this field for future studies on plants and their bioactive compounds.

## Introduction

Parkinson’s disease (PD) was first described by James Parkinson in 1817. The prevalence of PD is about 1% in people aged over 65 years. It begins between the ages of 40 and 70 and is very rare under the age of 20. If PD begins under the age of 20 years, it is referred to as young onset PD, which has a different pathology than other types of PD, is usually inherited, and is due to Wilson’s disease or Huntington’s disease. PD is more prevalent in men than in women by a ratio of 3:2 (Postuma et al. 2015).

The prevalence of PD is nearly 160 people per 100,000 population and its incidence is about 20 people per 100,000 population. The prevalence and incidence of the disease increase with age, so that at the age of 70 years, the prevalence is nearly 550 people per 100,000 and the incidence is 120 people per 100,000. Factors such as trauma, overwork, exposure to coldness, inflexible personality and stress are considered to be the predisposing factors; however, this has not yet been definitively proven (Tysnes and Storstein 2017).

### Etiology

The aetiology of PD is unknown. The disease, however, seems to be due to the dopamine depletion in the nigrostriatal pathway. Typically, intra-cytoplasmic inclusions, called Lewy bodies of dopamine neurons, are detected in PD. The degeneration of dopaminergic neurons in the substantia nigra (SN) compacta, followed by impaired release of dopamine in striatum, is a major cause of the disease; however, genetic factors have also been shown to be effective in the development of this disease. In 23% of cases, no genetic factor is identified. Several hypotheses have been raised for the death of dopaminergic cells in the SN compacta, such as mitochondrial complex defect associated with the electron transport chain, iron accumulation, protein accumulation, inflammatory immune responses along with environmental factors such as physical trauma and infection, liver cytochrome p450 dysfunction and increased formation of free radicals (Wang et al. 2015).

### Pathogenesis

PD is a neurodegenerative and progressive disease that is associated with various motor and debilitating disorders, including bradykinesia, muscle stiffness, resting tremor and imbalance. PD is pathologically characterized by slow and gradual degeneration of dopaminergic neurons in the SN compacta that leads to a decrease in the level of dopamine in the striatum, tailed nuclei and the putamen (Lees 2012). The progressive loss of dopaminergic neurons in the basal complexes is the most important pathological finding in the brain of patients with PD. Destruction of these neurons results in the reduction of dopamine neurotransmitter in this area. After 50–60% of dopaminergic neurons are degraded and dopamine levels in the striatum decrease by around 80–85%, the symptoms of the disease appear. The exact molecular mechanism of the degradation of dopaminergic neurons and the incidence of PD is unclear; however, studies have shown that oxidative stress and mitochondrial dysfunction probably play a key role in the pathogenesis of PD; the loss of nigrostriatal dopaminergic neurons and the presence of intracellular cytoplasmic proteins, i.e., Lewy bodies, are also involved. The cells are located in the nigrostriatal neurons in the SN pars compacta (SNpc) and are sent to putamen. The absence of these neurons, which typically contain small amounts of melanin, leads to depigmentation of SNpc (Lees 2012).

### Abnormal mitochondrial function and oxidative stress

Accumulating evidence suggests that in PD, the function of mitochondrial complex I partially decreases. Approximately 100% of molecular oxygen is consumed by the mitochondria during cellular respiration, and powerful oxidants, including hydrogen peroxide and superoxide radicals, are produced as a by-product. Reactive oxygen species (ROS) production increases by inhibiting mitochondrial complex I, which can produce toxic hydroxyl radicals or react with nitric oxide and produce peroxynitrites. These molecules can damage the nucleic acids, proteins and lipids by reacting with nucleic acids. One of these injuries can occur in the electron transport chain, which can lead to mitochondrial damage and the formation of ROS that, in turn, can increase the inappropriate folding of proteins (Gaki and Papavassiliou 2014).

**Figure 1.**
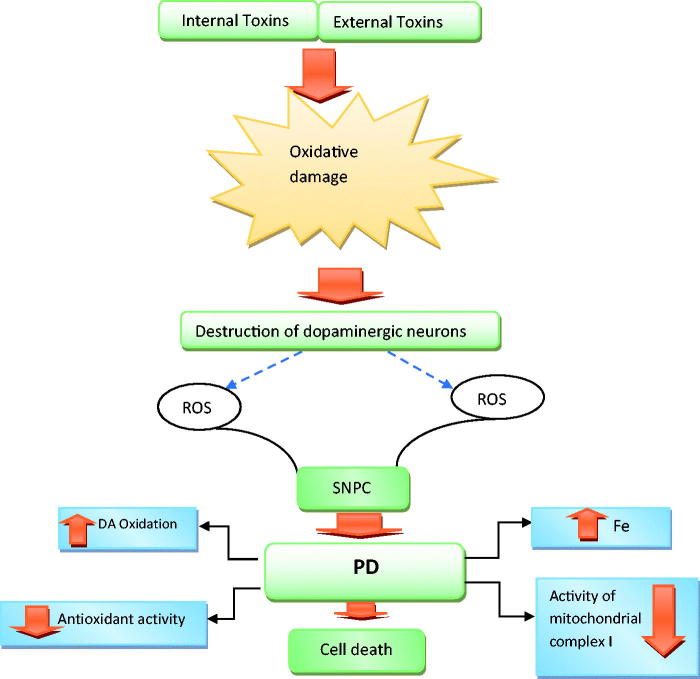
Factors that contribute to oxidative stress and ultimately neuronal cell death in PD.

Much research has also suggested that ROS plays a role in the degeneration of dopaminergic neurons in the brain tissues of PD patients (Figure 1). High levels of lipid peroxidation, glutathione depletion and increase‌ in protein oxidation are observed in the brain tissues of PD patients. Oxidation of dopamine leads to the formation of dopamine quinone that can directly alter proteins. In healthy people, there are mechanisms that prevent the cells from inappropriate folding and accumulation. For example, these proteins are harvested by lysosomal and ubiquitin-proteasome system, as well as certain chaperones can correct these foldings. It has been suggested that defective mitochondrial function causes the death of dopaminergic neurons at advanced ages. Oxidation of RNA and DNA of membrane proteins and lipids is one of the important factors for defective mitochondrial function. Oxidation changes the structure of many enzymes and thereby reduces their tendency to substrates or coenzymes, and thus their activity (Hwang 2013).

So far, many studies have been done on PD and the effects of various factors on the symptoms of the disease, the rate of its progression, and its treatment. Different methods have been used to induce PD in animal models and then the effects of various factors on the improvement of its symptoms have been evaluated. Animal models greatly help investigate pathogenic mechanisms and design therapeutic strategies in human diseases. Given that the pathogenesis of PD has not yet been well understood, it is important to use animal models to better understand the cause of PD and design the approaches to treat it. To induce PD in laboratory animals, certain compounds such as reserpine, methamphetamine, 6-hydroxydopamine, paraquat, maneb, rotenone and 3-nitrotyrosine also transgenic mice are used (Xu et al. 2002).

The most important approach to treat this disease is the use of precursors and other analogues of dopamine. There is a limitation in prescribing levodopa. Its chronic use is frequently associated with long-term motor impairment and decreased efficacy, requiring the increased dose of levodopa to reduce the complications of the disease. The function of levodopa depends on its enzymatic conversion to dopamine. Therefore, levodopa is usually prescribed in combination with carbidopa to be converted to dopamine by the DOPA decarboxylase (Antonini et al. 2010).

The general tendency to use herbal drugs and, in general, natural products in the world can be due to the side effects of chemical drugs on the one hand and environmental pollution on the other hand. Due to increasing prevalence of the nervous system disorders (Hasanpour-Dehkordi and Solati 2016; Hasanpour-Dehkordi et al. 2016; Solati and Lo’Bat Ja’Farzadeh 2016; Solati 2016, 2017) there is a growing tendency to the use of these plants and the revival of traditional medicine. Recent research has also reported promising results regarding the effects of herbal remedies in the treatment or prevention of various diseases, including Alzheimer’s disease (Rabiei et al. 2015; Rabiei and Setorki 2018), stroke (Rabiei et al. 2012; Rabiei and Rafieian-Kopaei 2014), depression (Rabiei et al. 2017, 2018) addiction (Rabiei et al. 2016) and many other conditions. The effects of medicinal plants and their active ingredients on the treatment of PD are presented in Tables 1 and 2.

**Table 1.** Plant active ingredients effective on Parkinson’s disease.

Name compound	Used model	Concentration	Effects	Chemical structures	References
Astragaloside IV	Cultures of primary nigral cells (PNCs)	50, 100 and 200 µM	1. Increased the level of TH and nitrite oxide synthase (NOS) immune reactivities 2. Protect dopaminergic neurons degeneration 3. Promoted neurite outgrowth and increased TH and NOS immune reactive of dopaminergic neurons		Chan et al. (2009)
Berberine	1. 6-OHDA induced cytotoxicity in PC12 cells 2. Unilateral 6-OHDA-lesioned rats	5, 10 and 30 µM	1. Depleted tyrosine hydroxylase-immuno positive cells in the substantia nigra 2. Decreased dopamine and norepinephrine levels in striatal regions		Kwon et al. (2010)
Baicalein	6-OHDA induced *in vitro* and *in vivo*	0.5, 5 μg/mL	1. Ameliorate SH-SY5Y cell apoptosis 2. Promote neurite outgrowth of PC12 cell 3. Attenuate muscle tremor 4. Increase tyrosine hydroxylase (TH)-positive neurons		Mu et al. (2009)
Caffeic acid phenethyl ester	*In vitro* (cerebellar granule neurons)	10 µm	1. Modulate the Ca^2+^-induced release of cyctochrome *c* 2. Inhibit caspase-3 activation 3. Blocks cell death		Noelker et al. (2005)
Carnosic acid	Rat induced by 6-OHDA	20 mg/kg	1. Improved the locomotor activity 2. Reduced the apomorphine-caused rotation 3. Reduced lipid peroxidation 4. GSH reduction 5. Increased the protein expression of c-glutamate-cysteine ligase catalytic subunit, superoxide dismutase, and glutathione reductase 6. Reduction of the Bcl-2/Bax ratio 7. Induction of caspase 3 cleavage 8. Induction of poly (ADP ribose) polymerase (PARP) cleavage		Wu et al. (2015)
Curcumin	Rat induced by 6-OHDA	80 mg/kg pretreatment	1. Decreased MDA, 2. Increased glutathione, GPx, glutathione reductase, SOD, catalase, tyrosine hydroxylase and D2 receptor binding in brain tissue		Khuwaja et al. (2011)
Gallic acid	Rat induced by 6-OHDA	50, 100 and 200 mg/kg	1. Increased the passive avoidance memory 2. Increased total thiol in brain tissue 3. Increased GPx 4. Decreased MDA levels in brain tissue		Mansouri et al. (2013)
Ellagic acid	Rat induced by 6-OHDA	50 mg/kg	1. Increased of stride-length 2. Decreased the contralateral rotations 3. Decreased TNF-α and IL-1β levels in brain tissue		Farbood et al. (2015)
Ginsenoside Rg1	(6-OHDA) induced neurotoxicity in human neuroblastoma SK-N-SH cells	0.01 µM	1. Increased survival 2. Rescue occurred on cell viability 3. Restore the up-regulation of Bax and down-regulation of Bcl-2 mRNA and protein expression 4. Attenuate 6-OHDA-induced apoptosis		Gao et al. (2009)
Quercetin	Rat induced by 6- OHDA	50 mg/kg	1. Increased dopamine 2. Decreased protein carbonyl content		Haleagrahara et al. (2013)
Desferrioxamine	Rat induced by 6-OHDA	50 mg/kg	1. Decreased protein carbonyl content 2. Increased dopamine, GSH and SOD levels		Haleagrahara et al. (2013)
Thymoquinone	Rat induced by 6- OHDA	5, 10 mg/kg	1. Improved turning behaviour 2. Decreased MDA levels 3. Increased activity of superoxide dismutase 4. Prevented loss of SNC (substantia nigra pars compacta) neurons		Sedaghat et al. (2014)
Tripchlorolide	(MPTP)-lesioned C57BL/6 mice	1 µg/kg	1. Increased level of dopamine in the substantia nigra and striatum		Hong et al. (2007)
Sulforaphane	mice induced by 6-OHDA	5 mg/kg	1. Improved motor coordination 2. Blocking DNA fragmentation and caspase-3 activation 3. Increased glutathione levels		Morroni et al. (2013)

**Table 2.** Medicinal plants effective on Parkinson’s disease.

Plant	Used model	Concentration	Effects	References
*Tinospora cordifolia*	Rat induced by 6-OHDA	200, 400 mg/kg	1. Increased the dopamine levels 2. Decreased iron asymmetry ratio 3. Decreased MDA levels 4. Increased mitochondrial complex I activity 5. Improved locomotor activity	Kosaraju et al. (2014)
Sesame seed oil (SO)	Mice induced by 6-OHDA	SO mix diet	1. Increased glutathione reductase (GR), glutathione-S-transferase (GST), glutathione peroxidase (GPx), catalase (CAT) and content of glutathione (GSH) and thiobarbituric acid reactive substance (TBARS) 2. Inhibit the activation of Nox2 and Cox2 3. Restored MnSOD expression	Ahmad S et al. (2012)
*Carthamus tinctorius* L.	(MPTP)-lesioned rat	70, 35 mg/kg	1. Improve behavioural performances 2. Suppression of α-synuclein overexpression or aggregation 3. Suppression of reactive astrogliosis	Ren et al. (2016)
*Chaenomeles speciosa*	*In vitro* and *in vivo* assays, Chinese hamster ovary (CHO) cells, rat induced by 6-OHDA (MPTP)-lesioned mice	250, 500, 1000 mg/kg	1. Increased tyrosine hydroxylase-positive neurons in the substantia nigra 2. Increased D8 cell viability 3. Time-dependently reduced abnormal turns in apomorphine-induced rotational turning	Zhao et al. (2008)
*Portulaca oleracea*	Rat induced by 6-OHDA	200, 400 mg/kg	1. Increase in crossings and rearing in open field test	Martins et al. (2016)
*Paeonia suffruticosa*	(MPTP)-lesioned mice	25, 50 mg/kg	1. Increased movement distance in the open field test 2. Increased total striatal dopamine 3. Attenuated the loss of dopaminergic neurons 4. Reversed down regulation Akt and the mitochondrial OXPHOS subunits	Kim et al. (2014)
*Mucuna pruriens*	Rat induced by 6-OHDA	40, 80, 120 mg/kg	1. Reduced risk for drug-induced dyskinesias 2. Increased nigrostriatal catecholamine content	Lieu et al. (2010)
*Hyoscyamus niger* seeds	Unilateral intrastriatal injection of rotenone in rat	125, 250, 500 mg/kg	1. Attenuated motor disabilities 2. Increased level of GSH content and GPX, SOD and CAT activities	Khatri and Juvekar (2015)
*Hibiscus asper* leaves	Rat induced by 6-OHDA	50, 100 mg/kg	1. Increased SOD, GPX and CAT activities, total GSH content 2. Reduced MDA level	Hritcu et al. (2011)
*Gynostemma pentaphyllum*	Rat induced by 6-OHDA	10, 30 mg/kg	1. Recovered the levels of dopamine, 3,4 dihydroxyphenylacetic acid, homovanillic acid and norepinephrine in striatum 2. Ameliorated the loss of TH-immunopositive neurons in substantia nigra	Choi et al. (2010)
*Ginkgo biloba*	Rat induced by 6-OHDA	50, 100, 150 mg/kg	1. Decreased rotation 2. Improved locomotor activity and muscular coordination 3. Increased GSH content 4. Decreased generation of TBARS 5. Increased SOD, CAT activities 6. Increase in the number of dopaminergic D2 receptors in striatum	Ahmad M et al. (2005)
*Fructus Alpiniaoxyphylla*	Zebrafish and PC12 cell models	20% solution	1. Restored dopaminergic (DA) neuron degeneration 2. Attenuated a deficit of locomotor activity 3. Increased the viability of 6-OHDA-treated PC12 cells 4. Attenuating cellular apoptosis	Zhang et al. (2012)
*Delphinium denudatum*	Rat induced by 6-OHDA	200, 400, 600 mg/kg	1. Decreased MDA levels 2. Increased GSH content 3. Increased SOD, CAT activities 4. Increased levels of dopamine	Ahmad M et al. (2006)
*Bacopa monniera* Linn	Rat induced by 6-OHDA	20, 40 mg/kg	1. Decreased MDA levels 2. Increased GSH content 3. Increased SOD, CAT activities	Shobana et al. 2012
*Althaea officinalis* L.	Rat induced by 6-OHDA	10 mg/kg	1. Attenuated rotational behaviour 2. Decreased MDA levels	Rezaei and Alirezaei (2014)
*Albizia adianthifolia*	Rat induced by 6-OHDA	150, 300 mg/kg	1. Improved working memory and reference memory 2. Attenuated the contralateral rotational asymmetry	Beppe et al. (2014)
*Valeriana officinalis*	Rotenone-induced apoptosis in human neuroblastoma SH-SY5Y cells	0.049, 0.098 and 0.195 mg/mL	1. Increase in cell viability	de Oliveria et al. (2009)
*Black tea*	Rat induced by 6-OHDA	1.5%	1. Recovery in d-amphetamine induced circling behaviour and spontaneous locomotor activity 2. Recovery indopamine (DA)-D2 receptor binding, striatal DA and 3-4-dihydroxy phenyl acetic acid (DOPAC) level 3. Decreased MDA levels 4. Increased GSH content 5. Increased SOD and CAT activities 6. Increased TH protein level and TH mRNA expression in substantianigra	Chaturvedi et al. (2006)
*Panax ginseng*	Rat received β-sitosterol β-*D*-glucoside	100 mg/kg/d	1. Reduced dopaminergic cell loss, microgliosis, and accumulation of α-synuclein aggregates	Van Kampen et al. (2014)
Safflower	Mouse induced with 1-methyl-4-phenyl-1,2,3,6-tetrahydropyridine-	35, 70 mg/kg/d	1. Reversed the decreased protein expression of tyrosine hydroxylase, dopamine transporter and DJ-1 2. Increased dopamine levels 3. Decreased acetylcholine levels	Ablat et al. (2016)
*Hypericum perforatum*	Rat induced by 6-OHDA	200 mg/kg/d	1. Attenuated apomorphine-induced rotational behaviour, 2. Decreased the latency to initiate and the total time on the narrow beam task 3. Decreased MDA levels 4. Increased catalase activity	Kiasalari et al. (2016)
*Oxalis corniculata l.*	C57 male mice MPTP administration	250, 500 mg/kg	1. Decreased SOD activity 2. Increased catalase activity	Aruna et al. (2016)

## Discussion

Medicinal plants have been discovered by different nations across the world thousands of years ago. These plants are used in many communities and countries for centuries due to their safety, efficiency, acceptability and comparably fewer side effects than chemical drugs (Alok et al. 2013). Medicinal plants have long been known throughout the world for their unique and valuable benefits. Research has also shown that these plants have a special status in the health and well-being of various communities due to the antioxidant effect of phenolic compounds identified in them, in addition to their economic value. Therefore, the global approach is to identify new plant species and their active ingredients. Today, plants play an important role in the therapeutic approaches and new drugs used for diseases. Therefore, the demand for new oral medicines without side effects continues. In this study, the therapeutic effects of medicinal plants and their compounds on the treatment of PD observed *in vivo* and *in vitro* were reviewed. Although most of the herbal extracts and their active ingredients have been studied in PD models *in vivo*, some of them have so far been investigated only in cell models (Noelker et al. 2005; Chan et al. 2009; Gao et al. 2009).

To date, a large number of medicinal plants and their active ingredients have been reported to prevent and treat PD. Most studies have focused on antioxidant (Ahmad M et al. 2005, 2006; Chaturvedi et al. 2006; Hritcu et al. 2011; Khuwaja et al. 2011; Ahmad S et al. 2012; Shobana et al. 2012; Mansouri et al. 2013; Khatri and Juvekar 2015; Wu et al. 2015), anti-inflammatory (Farbood et al. 2015) and antiapoptotic (Gao et al. 2009; Morroni et al. 2013; Kim et al. 2014) properties of these plants.

A number of studies have demonstrated the protective effects of tea polyphenols on brain damage in different animal models of PD. Studies have used either a single compound such as EGCG, or a combination of extracts from tea. Individual EGCG and black tea polyphenol extracts were observed to mitigate striatal dopamine depletion and SN dopaminergic neurons loss after being administered for the long term to rats or mice receiving the parkinsonism-inducing neurotoxins, e.g., 6-OHDA and 1-methyl-4-phenyl 1,2,3,6-tetrahydropyridine (MPTP) (Chaturvedi et al. 2006).

The aqueous extract of *Albizia adianthifolia* W. Wight (Leguminosae) leaves has antioxidant potential and can help manage neurological abnormalities due to PD. The HPLC analysis showed that apigenin is the main compound among flavones, and therefore the cause of the cognitive-enhancing impacts in the 6-OHDA-lesion rodent model of PD. Apigenin and similar compounds stimulate neurogenesis in adults both *in vivo* and *in vitro* via promoting neuronal differentiation, and also promote learning and memory according to Morris water task (Beppe et al. 2014).

At present, the pathogenesis of PD is attributed to the formation of ROS and the onset of oxidative stress, leading to damage to the SN compacta, particularly changes in the brain’s iron content, mitochondrial dysfunction, changes in antioxidant defence system (especially reduction in superoxide dismutase and glutathione [GSH]) and oxidative damage to fats, proteins and DNA. The loss of GSH is related to Lewy body disease (Figure 1). GSH may be the most important biochemical marker for loss of nigral cells. The depletion of GSH may not be the only reason for damage to nigral neurons, but may increase the susceptibility to exposure to toxic or free radicals (Hwang 2013). Most of the medicinal plants and active ingredients reported in this paper increase the levels of glutathione, superoxide dismutase and catalase in the brain, and thus exert neuroprotective effects. Evidence indicates the role of inflammation in the pathogenesis of PD. In patients with PD, the active microglia increase in the striatum and the SN. Microglial cells, which are macrophages in the brain, respond under numerous unfavourable conditions through fast hypertrophic proliferation and expression of a number of cytokines. Active microglia upregulate cell surface markers, such as macrophage antigen complex 1, produce a variety of pro-inflammatory cytokines. A number of cytokines, including IL-1, IL-6 and TNF-α, contribute to the inflammation process (Niranjan 2014).

Certain active ingredients of plants, such as ellagic acid, reduce rotation in rats with 6-OHDA-induced PD due to reduced inflammation by lowering the levels of IL-1β and TNF-α in the brain tissue (Farbood et al. 2015). Since a plant extract contains a wide range of compounds, the effects of synergistic reaction of two or more compounds is higher than those of a single substance (Williamson 2001). Synergistic effects may be responsible for the therapeutic effects of many plant-based products. Therefore, herbal extracts can be used to treat PD due to various bioactive ingredients (Wang et al. 2008).

## Conclusions

We sought to present a summary of current experimental evidence on anti-Parkinsonian activities of medicinal plants and their active ingredients. We found that different natural compounds and herbal extracts exhibit various anti-Parkinsonian activities. Taken together, various PD neurotoxic models are a good basis for discovering anti-Parkinsonian drugs, and herbal drugs can be used to develop new drugs for PD. However, the efficacy of plant extracts and their active ingredients in PD models should be further investigated in future empirical studies. In addition, the active ingredients and action mechanisms of herbal extracts remain to be adequately explained.
